# Emotional regulation as factor of commitment to paralympic sports

**DOI:** 10.1192/j.eurpsy.2021.1230

**Published:** 2021-08-13

**Authors:** A. Yavorovskaya, I. Polikanova, Y. Semenov, S. Leonov, E. Rasskazova

**Affiliations:** 1 Faculty Of Psychology, Lomonosov Moscow State University, Moscow, Russian Federation; 2 Clinical Psychology, Moscow State University, Moscow, Russian Federation

**Keywords:** Paralympic sports, Emotional Regulation, Commitment to sports

## Abstract

**Introduction:**

Commitment to sports is especially important for well-being in people with disabilities (Malm et al., 2019), although mental health problems are common among Paralympic athletes (Hunt et al., 2019). Importance of representations of and regulation in sport situations was supported for different kind of sports (Moran, 1993, Suinn R., 1982, Hardy et al., 1996) but Paralympic ones.

**Objectives:**

The aim was to reveal aspects of psychological regulation important for achievement and longer commitment to Paralympic sports comparing to non-Paralympic sports.

**Methods:**

51 athletes from Paralympic sports (49.1% candidates and masters of sports) filled Questionnaire of Image Representations of Professional Activity of Athletes (Leonov et al., 2020) measuring general importance and self-appraisals of different aspects of image representation and regulation in sport activities: control of temporal, spatial, informational, technical and tactical, energetic aspects, game intelligence, motivational, emotional and social aspects (Cronbach’s alphas .61-.89). Data were compared to 399 athletes without disabilities (48.4% candidates and masters of sports).

**Results:**

Comparing to athletes without disabilities, Paralympic athletes higher appraise general importance and their capacities for emotion regulation during sport situation (t=2.26-3.35, p<.01). High-level Paralympic atheletes report marginally better emotion regulation (t=1.74, p<.10). Longer experience in sport in Paralympic athletes is associated with better representations of spatial and social aspects of sport situations and better emotion regulation (r=.25-.29, p<.05).

**Conclusions:**

Data suggest that improvement of emotion regulation in sport situation in Paralympic athletes could be helpful for longer participation and better achievement. Research is supported by the Russian Science Foundation, project No. 19-78-10134.

**Conflict of interest:**

Research is supported by the Russian Science Foundation, project No. 19-78-10134

